# Genetic or Pharmaceutical Blockade of Phosphoinositide 3-Kinase P110δ Prevents Chronic Rejection of Heart Allografts

**DOI:** 10.1371/journal.pone.0032892

**Published:** 2012-03-30

**Authors:** Huijun Ying, Hongmei Fu, Marlene L. Rose, Ann M. McCormack, Padmini Sarathchandra, Klaus Okkenhaug, Federica M. Marelli-Berg

**Affiliations:** 1 Department of Immunology, Division of Medicine, Imperial College London, Hammersmith Campus, London, United Kingdom; 2 National Heart & Lung Institute, Division of Medicine, Imperial College London, Harefield Hospital, London, United Kingdom; 3 Laboratory of Lymphocyte Signalling and Development, Babraham Institute, Cambridge, United Kingdom; Bristol Heart Institute, University of Bristol, United Kingdom

## Abstract

Chronic rejection is the major cause of long-term heart allograft failure, characterized by tissue infiltration by recipient T cells with indirect allospecificity. Phosphoinositol-3-kinase p110δ is a key mediator of T cell receptor signaling, regulating both T cell activation and migration of primed T cells to non-lymphoid antigen-rich tissue. We investigated the effect of genetic or pharmacologic inactivation of PI3K p110δ on the development of chronic allograft rejection in a murine model in which HY-mismatched male hearts were transplanted into female recipients. We show that suppression of p110δ activity significantly attenuates the development of chronic rejection of heart grafts in the absence of any additional immunosuppressive treatment by impairing the localization of antigen-specific T cells to the grafts, while not inducing specific T cell tolerance. p110δ pharmacologic inactivation is effective when initiated after transplantation. Targeting p110δ activity might be a viable strategy for the treatment of heart chronic rejection in humans.

## Introduction

Chronic rejection is the main cause of late heart allograft failure and the leading cause of death in patients surviving more than 1 year after transplantation [1], [2]. Prominent features of chronic heart graft rejection include proximal coronary artery vasculopathy, occlusion, and eventually loss of cardiac function [1]–[3]. These lesions are associated with substantial parenchymal infiltration by T cells [4]. Host immunity – particularly indirect alloresponses mediated by CD4^+^ T cells, as well as antibody-mediated immune responses – to processed fragments of donor major histocompatibility antigens (MHC) and to minor histocompatibility antigens (mHC) have been linked to the development of chronic heart allograft rejection [5]–[15].

Besides antigen-induced activation, the development of immune responses requires active mechanisms of recruitment of antigen-specific primed T cells into antigenic sites. We and others have shown that T cell receptor (TCR) engagement by antigen-presenting endothelium leads to the migration of antigen-specific memory T cells to non-lymphoid antigen-rich target tissue following priming [16]–[20]. This effect is required for the development of a number of T cell-mediated diseases in mice [20]–[22]. The effect of TCR ligation on T lymphocyte motility is likely to engage signaling pathways linking TCR triggering to the cytoskeleton. Class IA phosphoinositide 3-kinases (PI3Ks) are a family of p85/p110 heterodimeric lipid kinases that generate second messenger signals (e.g., PIP3) downstream of tyrosine kinases, thereby controlling various cell functions, including motility. PI3K p110δ subunit expression is restricted to hematopoietic cells [23]. Following TCR triggering, p110δ is recruited by adaptor proteins [24], [25]. Previous studies have shown that mice expressing a catalytically inactive form of p110δ (P110δ^D910A^) display attenuated T cell-mediated immunity, although p110δ^D910A^ mice can be primed against nominal antigens [26]. We have recently shown that, while chemotaxis and constitutive trafficking of memory T lymphocytes with impaired p110δ activity are unaffected, these T cells are not susceptible to TCR-mediated T cell recruitment to antigenic sites, which they fail to infiltrate [21].

In this study, we have investigated the effect of PI3K p110δ inactivation on the development of chronic rejection in a murine model of HY-mismatched heart allograft. We show that the establishment of chronic rejection is significantly attenuated in mice lacking p110δ activity in the absence of any additional immunosuppressive treatment. The therapeutic effects of p110δ inhibition correlated with impaired localization of HY-specific memory T cells to the allografts, but did not induce T cell tolerance. Importantly, PI3K p110δ pharmacologic inactivation is effective even when initiated after transplantation. We propose that selective PI3K p110δ inhibitors can be developed into an effective therapeutic tool to control chronic heart allograft rejection.

## Results

### Genetic abrogation of PI3K p110δ–signaling prevents T-cell-mediated chronic heart allograft rejection

PI3K p110δ has been shown to play a critical and non-redundant role in the activation and differentiation of naive T cells [27]. We therefore sought to investigate the effect of inhibition of PI3K p110δ signaling on the development of immune-mediated mechanisms of chronic heart allograft rejection. A well-established model involving transplantation of HY-mismatched heart allografts, in which grafts develop pathological features of chronic rejection over time [28], was adapted for this study. Development of pathology in this model is strictly T cell-dependent, antibody-independent [29], and occurs without cessation of the heartbeat [28]. For this reason, histopathologic assessments, rather than survival time points, are provided.

**Figure 1:**
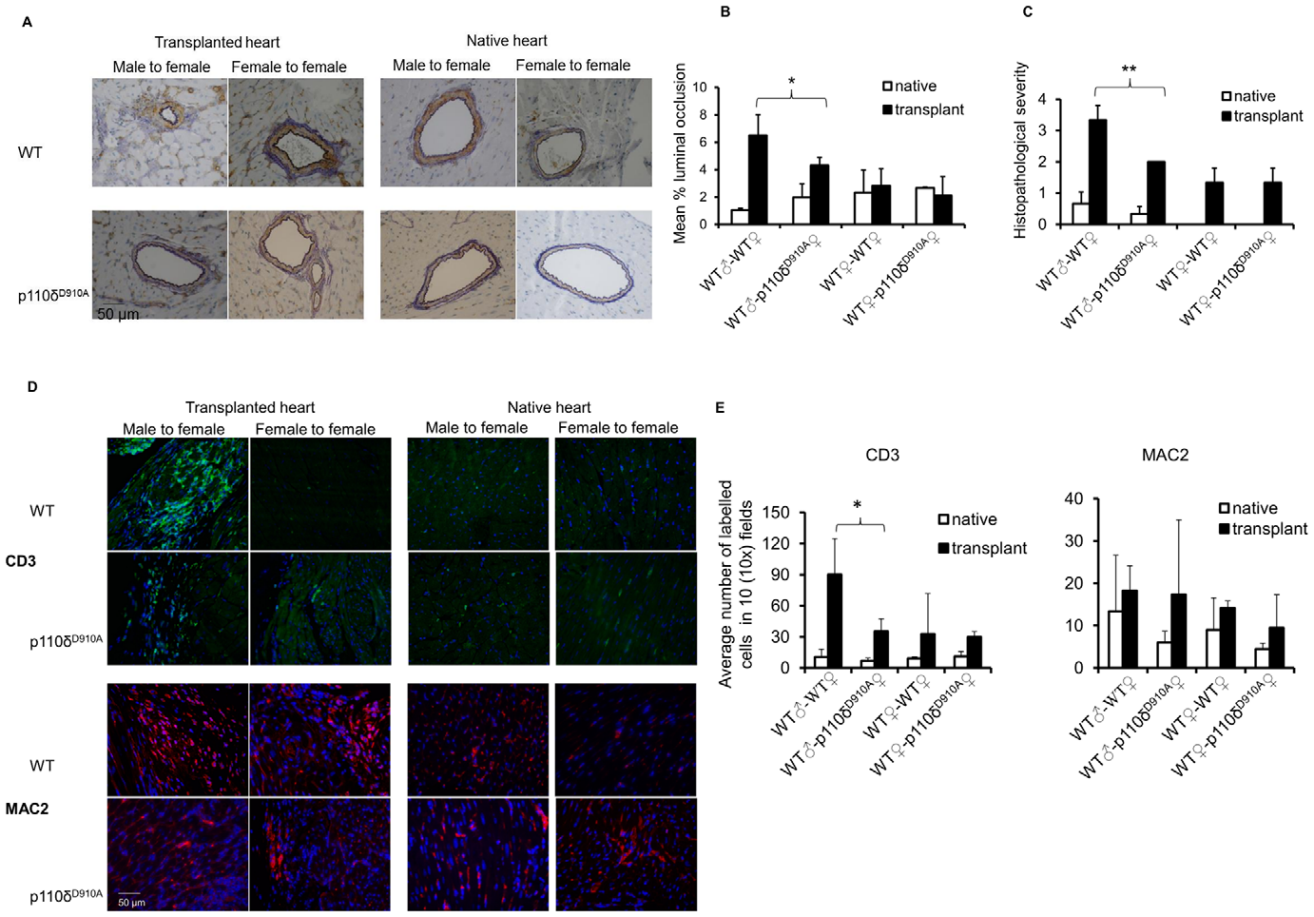
Genetic abrogation of PI3K p110δ signaling prevents T-cell-mediated chronic heart allograft rejection. Recipient female WT and p110δ^D910A^ mutant mice received either male or female heart. Both transplanted and native hearts were harvested 23 days after transplant. (A) Tissue sections were stained with Miller's elastin followed by immunoperoxidase staining for SMCs using rabbit monoclonal antibody to mouse SMC alpha -actin, then counterstained with hematoxylin. Luminal occlusion was evaluated by tracing the cross-section of each vessel's internal elastic lamina and lumen using software in two transverse sections per graft. Each panel shows a representative tissue image. Magnification: 20x. (B) The mean percentage luminal occlusion ± SD observed in 3 samples obtained from each recipient (at least 3 animals/group) is shown. *p<0.03 (C) The mean histopathological scores ± SD of transplanted hearts stained with HE observed in 3 samples obtained from each recipient (at least 3 animals/group) is shown. 0, no inflammation; 1, light focal lymphohistocytic infiltrate; 2, moderate focal lymphohistocytic infiltrate with myocardial involvement; 3, moderate to severe inflammation with focal vasculopathy and myocyte degeneration; 4, severe inflammation, vasculopathy and myocardial fiber loss. **p<0.01. (D) Tissue sections were stained with FITC-labelled anti-CD3 antibody and PE-labelled anti-MAC2 antibody. Each panel shows a representative tissue image. Magnification: 20x. (E) The mean T cell or macrophage infiltration ± SD observed in 3 samples obtained from each recipients (at least 3 animals/group) is shown. *p<0.05.

Recipient female WT and p110δ^D910A^ mutant mice (bearing an inactive form of p110δ [26]) received either male (antigenic) or female (non-antigenic control) WT hearts. 23 days after transplant, both transplanted and native hearts were harvested and stained with hematoxilin/eosin (HE, representative images in Figure S1), and Miller’s elastin combined with SMC alpha actin immuno-staining (Figure 1A). This time point was selected based on previous monitoring of pathology development (data not shown) and license constraints.

As it is shown in Figure 1, heart allografts placed into p110δ^D910A^ female recipients were protected from the development of vasculopathy as assessed by histopathologic criteria. Co-staining of elastine end SMC alpha actin revealed early signs of vasculopathy (narrowing of the lumen and perivascular proliferation of SMC [30]) in female WT recipient of male hearts, which was inhibited in p110δ^D910A^ female recipients (Figure 1A–B). HE staining of the tissues revealed severe inflammatory lesions in WT female recipients of male hearts, which were significantly attenuated in p110δ^D910A^ female recipients (Figure 1C and Figure S1). Female graft and native hearts were free of disease.

Graft infiltration by T cells and macrophages was assessed by immunostaining with FITC-conjugated anti-CD3 and PE-conjugated anti-MAC2 antibodies. As shown in Figure 1D (representative tissue images from each group) and Figure 1E (mean T cell infiltration ± SD), T cell infiltration of male heart grafted into female p110δ^D910A^ mutants was significantly reduced compared with that observed in transplanted male heart grafted into WT female recipients. No difference in T cell infiltration of either female-derived heart grafts or native hearts was observed. Interestingly, no significant difference in the number of infiltrating macrophages was observed in any of the combinations tested. Although p110δ has been shown to affect B cell chemotaxis [31], these data suggest that T cell p110δ activity is selectively targeted in this model, in which the development of chronic rejection is strictly T cell-dependent and B cell-independent [28].

### PI3K p110δ inhibition does not induce T cell tolerance

**Figure 2:**
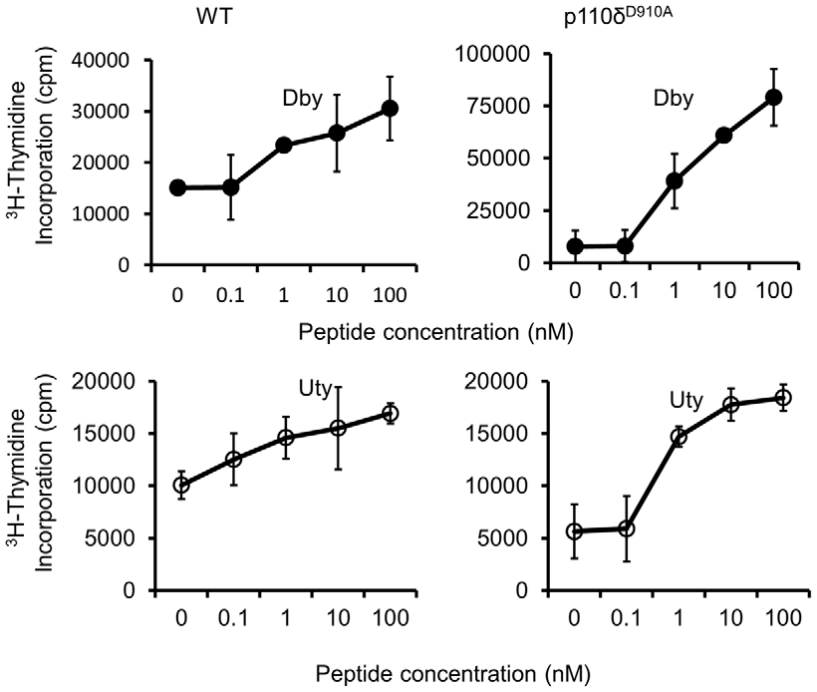
Loss of PI3K p110δ activity does not induce T cell tolerance . Recipient female WT and p110δ^D910A^ mutant mice received male WT transplanted heart. 60 days after transplant, splenocytes from either recipient WT or p110δ^D910A^ mutant mice were incubated with different concentrations of *Dby* and *Uty* HY peptide epitopes for 48 hours, followed by pulsing with [^3^H] thymidine to assess T cell proliferation.

PI3K p110δ has been reported to contribute to T-cell activation and differentiation [23], [26]. We therefore sought to investigate whether the lack of PI3K p110δ activity led to loss of responsiveness by HY-specific T cells following transplantation. Splenocytes from female WT and p110δ^D910A^ recipients were harvested 23 days after heart transplantation. T cells were cultured with increasing concentrations of *Dby* or *Uty* HY epitope peptides for 48 hours, followed by assessment of thymidine incorporation. As shown in Figure 2, both WT and p110δ^D910A^ T cells proliferated in response to HY-derived peptides, suggesting that antigen-specific T cell responsiveness was maintained in mice which did not develop chronic rejection as a result of PI3K p110δ inactivation.

### PI3K p110δ is required for male heart graft infiltration by HY-specific T cells

Antigen presentation by graft endothelium has previously been shown to be instrumental to T cell infiltration and rejection of HY-mismatched allografts [21], [28].

Given that PI3K P110δ inactivation did not lead to antigen-specific T cell tolerance, we sought to investigate whether the protective effect of abrogation of PI3K p110δ signaling selectively prevented antigen-dependent T cell recruitment to HY-mismatched heart graft. C57BL/6 female mice received a syngeneic male (HY-mismatched) or female (non-antigenic) heart transplant. On day 15 post-heart-grafting (i.e. once a memory T cell response is physiologically established[32]), PKH26-labelled HY-specific H2-A^b^-restricted CD4^+^ WT and CFSE-labeled HY-specific H2-A^b^-restricted p110δ^D910A^ CD4^+^ T cells (10^7^/mouse) were injected *i.v*. into female recipients of a WT male heart. The presence of labeled T cells in both transplanted and native hearts was analyzed 24 hours later by wide-field fluorescence microscopy.

**Figure 3:**
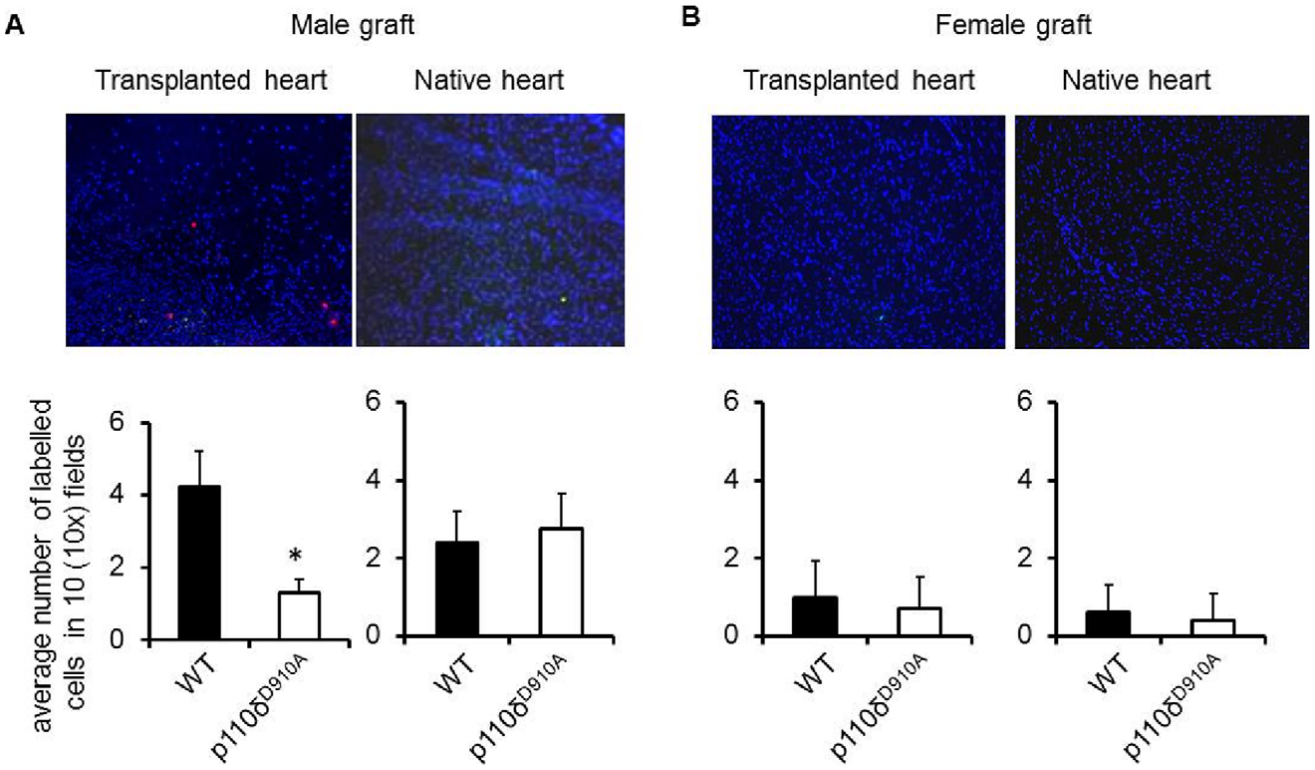
PI3K p110δ is required for heart graft infiltration by antigen-specific T cells . PKH26-labelled HY-specific H2-A^b^-restricted CD4^+^ WT and CFSE labelled HY-specific H2-A^b^-restricted p110δ^D910A^ T cells were injected *i.v*. into female mice transplanted with either male (A) or female (B) syngeneic heart. T cell localization in the transplanted heart and native heart were assessed 24 hours later by wide-field fluorescence microscopy. Tissue infiltration was quantified by randomly selecting ten ×10-magnified fields from tissue samples obtained from each mouse from all the experimental groups and assessing the number of fluorescent cells in each field. Each panel shows a representative tissue image. The mean T cell infiltration ± SD observed in samples from at least 3 animals is shown. Magnification: 10x. *p<0.05.

As shown in Figure 3, WT T cells promptly infiltrated male (A) but not female-derived (B) heart grafts, while p110δ^D910A^ T cell localization to male transplanted hearts was significantly reduced (A). These results demonstrated that PI3K p110δ activity is required for efficient access of HY-specific T cells to male heart grafts. Interestingly, some T cell infiltration was observed in native hearts of both WT and p110δ^D910A^ recipients of male hearts, possibly driven by non-specific inflammation induced by the allograft, which was nevertheless unable to induce pathology.

### PI3K p110δ is not required for constitutive trafficking by memory HY-specific T cells

We have previously suggested that lack of p110δ activity specifically affects antigen-driven migration, but not constitutive memory T cell trafficking [21]. The chronic rejection model allowed us to investigate whether this observation holds true in the presence of inflammation. We therefore assessed the migration of HY-specific T cells in sites of constitutive homing in C57BL/6 female recipients of a syngeneic male heart. On day 15 post-grafting, PKH26-labelled HY-specific H2-A^b^-restricted CD4^+^ WT and CFSE labeled HY-specific H2-A^b^-restricted p110δ^D910A^ T cells (10^7^/mouse) were injected *i.v*. into female recipients of a WT male heart. T cell localization in the liver, kidney, lymph node, spleen and gut were assessed 24 hours later by wide-field fluorescence microscopy.

**Figure 4:**
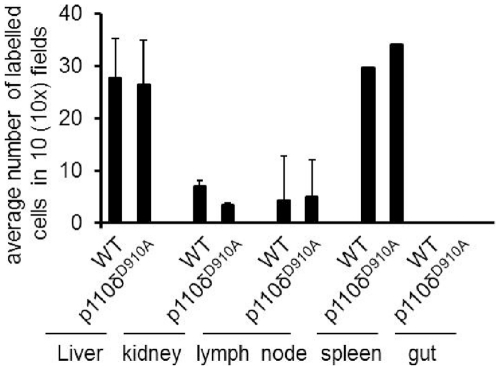
PI3K p110δ is not required for constitutive trafficking by antigen-specific T cells . PKH26-labelled HY-specific H2-A^b^-restricted CD4^+^ WT and CFSE labelled HY-specific H2-A^b^-restricted p110δ^D910A^ T cells were co-injected *i.v*. into female mice recipient of syngeneic hearts. T cell localization in the liver, kidney, lymph node, spleen, gut and native heart were assessed 24 hours later by wide-field fluorescence microscopy. To minimize the effect of arbitrary choice of field, tissue infiltration was quantified by randomly selecting ten×10-magnified fields from tissue samples from at least 3 animals and assessing the number of fluorescent cells in each field. Each panel shows a representative tissue image. The mean T cell infiltration ± SD observed in samples from at least 3 animals is shown.

As shown in Figure 4, both WT and p110δ^D910A^ T cells re-circulated normally and could be detected in the liver, kidney, lymph node, and spleen of recipient mice in similar numbers. Notably, WT and p110δ^D910A^ T cells displayed similar patterns of distribution within the various organs and localized in the liver and kidney in a scattered pattern, while they clustered in restricted areas in lymph nodes. Some T cell infiltration was observed in native hearts, irrespective of p110δ activity. These observations further confirm that p110δ signaling selectively regulates T cell migration to tissues that do express cognate antigen and it is not required for constitutive trafficking of T cells.

### Pharmacologic inhibition of PI3K p110δ in HY-mismatched heart allograft recipients inhibits the development of chronic heart allograft rejection

We have previously shown that PI3K p110δ inhibition selectively targets memory T cell trafficking [21]. This opens the possibility that targeting PI3K p110δ might be effective in a therapeutic regime. We therefore investigated whether pharmacologic inactivation of PI3K P110δ delivered after transplantation at a time when the immune response is already established could prevent the development of chronic rejection. Recipient female WT mice received either syngeneic male or female heart grafts. After 7 days, the selective PI3K p110δ inhibitor IC87114 (60mg/kg/day) or vehicle control was administered i.p. daily for 15 days. Mice were sacrificed 24 hours after the last treatment (day 23). Both transplanted and native hearts were harvested for analysis.

**Figure 5:**
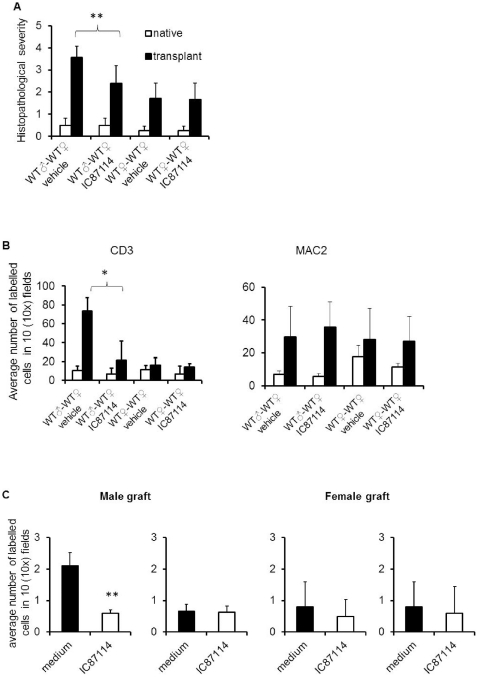
Pharmacologic inhibition of PI3K p110δ inhibits chronic heart rejection by preventing T cell access to the graft . Recipient female WT mice received either syngeneic male or female heart grafts. After 7 days, the selective PI3K p110δ inhibitor IC87114 (60mg/kg/day) or vehicle control were injected i.p daily for 15 days. Mice were sacrificed 24 hours after the last treatment (day 23). (A) The mean histopathological scores ± SD of transplanted hearts stained with HE observed in 3 samples obtained from each recipient (at least 3 animals/group) is shown. 0, no inflammation; 1, light focal lymphohistocytic infiltrate; 2, moderate focal lymphohistocytic infiltrate with myocardial involvement; 3, moderate to severe inflammation with focal vasculopathy and myocyte degeneration; 4, severe inflammation, vasculopathy and myocardial fiber loss. **p<0.01 (B) Both transplanted and native hearts were harvested and tissue sections were stained with either FITC-labelled anti-CD3 antibody or PE-labelled anti-MAC2 antibody. The mean T cell infiltration ± SD observed in samples from at least 3 animals is shown. Filled bar: transplanted heart; Non-filled bar: native heart. *p<0.05. (C) PKH26-labelled HY-specific H2-A^b^-restricted CD4^+^ WT and CFSE labelled CD4^+^ WT treated with IC87114 were injected *i.v*. into female mice with male syngeneic heart transplantation. T cell localization in the transplanted heart and native heart were assessed 24 hours later by wide-field fluorescence microscopy. Tissue infiltration was quantified by randomly selecting ten ×10-magnified fields from tissue samples from at least 3 animals and assessing the number of fluorescent cells in each field. Each panel shows a representative tissue image. The mean T cell infiltration ± SD observed in samples from at least 3 animals is shown. **p<0.01.

As it is shown in figure 5A, histological analysis showed that treatment with PI3K p110δ inhibitor IC87114 prevented the development of pathological signs of chronic rejection (representative images are depicted in Figure S2 panel A). Similarly, T cell infiltration of male heart grafted into WT female mice treated with IC87114 was significantly reduced compared to that observed in transplanted male heart grafted into WT recipient female mice treated with vehicle control (Figure 5B and Figure S2 panel B). No significant differences in T cell infiltrates were observed in either female-to-female transplanted heart grafts or native hearts. Macrophage infiltrates were often observed, but were of similar magnitude in any donor to recipient combination tested, irrespective of the development of pathology.

Similarly to what we observed in p110δ^D910A^ recipients of male hearts, T cells obtained from WT female recipients treated with or without IC87114 responded equally well to HY-derived *Dby* and *Uty* epitopes, suggesting that IC87114 treatment did not affect T cell responsiveness (Figure S3).

Finally, we sought to establish whether, like genetic inactivation, pharmacological inhibition of P110δ selectively affects localization of specific T cells to the heart allograft. Recipient female WT mice received either syngeneic male or female heart grafts. On day 15 post-heart-grafting, PKH26-labelled HY-specific H2-A^b^-restricted CD4^+^ WT and CD4^+^ WT treated with IC87114 (5μM, 1 hour at 37°C) (10^7^/mouse) were injected *i.v*. into female mice recipients of syngeneic male or female-derived hearts. The presence of labeled T cells in both transplanted and native hearts were analyzed 24 hours later by wide-field fluorescence microscopy. As shown in Figure 5C, untreated T cells promptly localized to male heart grafts, unlike CD4^+^ WT T cells treated with IC87114. No difference in T cell infiltration was observed in female heart grafts or native hearts. These results suggest that pharmacologic PI3K p110δ inactivation is effective at inhibiting access of activated HY-specific T cells to mHC-mismatched heart grafts in a therapeutic regimen.

### PI3K p110δ inactivation does not prevent rejection of HY-mismatched skin

**Figure 6:**
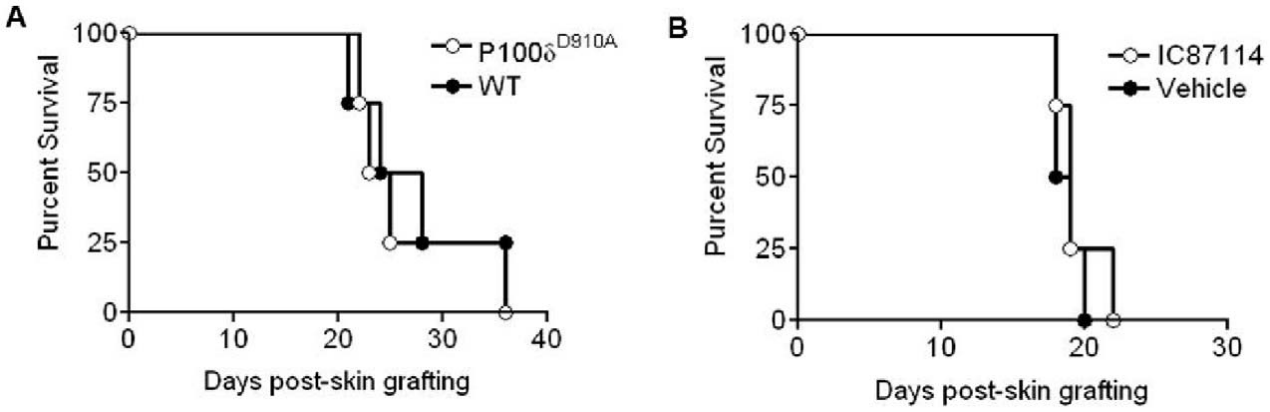
PI3K p110δ inactivation does not prevent rejection of HY-mismatched **skin**. (A) Recipient female WT or p110δ^D910A^ mice received WT male skin grafts. Graft survival was monitored daily for up to 4 weeks. (B) Recipient female WT mice received male skin grafts. 7 days after transplant, PI3K p110δ inhibitor IC87114 at 60mg/kg/day or vehicle control were injected i.p daily until the grafts were rejected.

Immune-mediated rejection of vascularized (heart) and non-vascularized (skin) allografts relies upon different mechanisms [21], [28], [33], [34]. Having shown that PI3K p110δ inactivation either by genetic mutation or pharmacological inhibition resulted in inhibition of chronic rejection of male heart grafts, we assessed the effect of PI3K p110δ inactivation in a model of HY-mismatched skin transplantation. Recipient female WT or p110δ^D910A^ mice received WT male skin grafts, and the occurrence of rejection was monitored. Alternatively, WT female recipients of a syngeneic male skin graft were treated daily with PI3K p110δ inhibitor IC87114 at 60mg/kg/day or vehicle control 7 days after transplantation until rejection. As it is shown in Figure 6A and 6B, neither genetic nor pharmacological inhibition of PI3K p110δ activity led to enhanced skin graft survival. Splenocytes from all groups proliferated equally well to both the HY Dby and Uty epitopes (Figure S4).

We further investigated the ability of HY-specific T cells to infiltrate skin grafts. Both male and female skins were grafted onto female recipient mice. On day 20 post-grafting, PKH26-labelled HY-specific H2-A^b^-restricted CD4^+^ WT and CFSE labelled HY-specific H2-A^b^-restricted p110δ^D910A^ T cells were injected *i.v*. into recipient mice. In parallel experiment, HY-specific WT T cells were treated with IC87114 (5μM, 1 hour at 37°C) (10^7^/mouse) or vehicle (DMSO) before injection. The presence of labelled T cells in skin grafts were analyzed 24 hours later by wide-field fluorescence microscopy.

**Figure 7:**
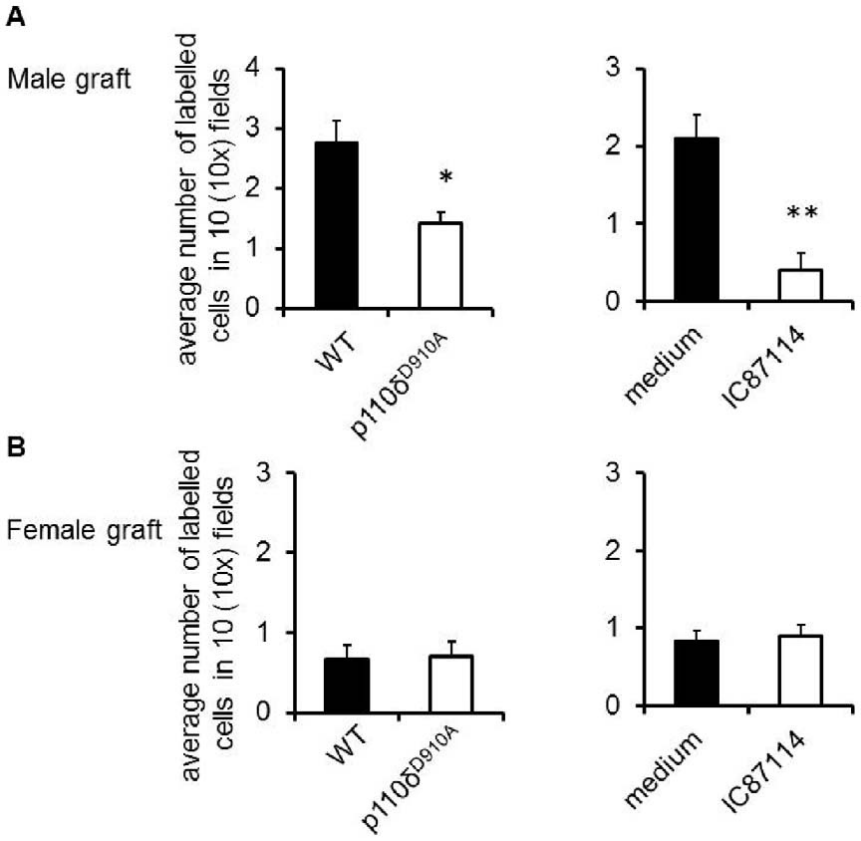
PI3K p110δ inactivation reduces skin graft infiltration by antigen-specific T cells . On day 20 post-skin grafting, PKH26 labelled HY-specific 10x 10^6^ WT and CFSE labelled 10^7^ P110δ*^D910A^* T cells or WT T cells treated with PI3K p110δ inhibitor IC87114 (5µM for 1 hour at 37°C) were injected *i.v*. into recipient mice. T cell localization in male skin (A) and female skin (B) was assessed 24 hours later by wide-field fluorescence microscopy. Tissue infiltration was quantified by randomly selecting ten ×10-magnified fields from at 3 tissue samples from at least 3 animal groups and assessing the number of fluorescent cells in each field. The mean T cell infiltration ± SD observed in samples from at least 3 animals is shown. *p<0.05, **p<0.01.

As shown in Figure 7, inhibition of PI3K p110δ activity either by genetic mutation or pharmacological inhibition prevented HY-specific T cell infiltration to male skin grafts. These results suggest that rejection of non-vascularized skin grafts relies upon inflammatory mechanisms other than graft infiltration by antigen-specific T cells.

## Discussion

In this study we have investigated the effect of genetic or pharmacologic inactivation of PI3K p110δ on the development of chronic allograft rejection in a murine model of mHC (HY)-mismatched heart allograft. We show that inhibition of PI3K p110δ activity significantly reduces the development of chronic rejection by inhibiting memory T cell access to the allograft.

Following activation, efficient memory T cell localization to antigen-rich sites requires a sequence of signals, mostly delivered by the endothelium, which include tissue-selective homing interactions such as those mediated by adhesion molecule and chemokine ligand to reach and access target tissue [28], [35]. We and others have shown that efficient recruitment of antigen-specific T cells into antigen-rich sites with promiscuous adhesion/chemokine receptor/ligand pairs (such as the heart) is optimized by TCR triggering of specific T cells by antigen-presenting endothelium [19], [20], [28], [36]. Importantly, this effect has been shown to support the localization of effector T cells to mHC-mismatched heart allograft leading to chronic rejection [28]. We have also reported that antigen-dependent recruitment by the endothelium strictly relies upon PI3K p110δ activity, which is initiated upon TCR triggering by MHC:peptide complexes displayed on the endothelial surface[21].

In our study, the prevention of pathological inflammation leading to chronic rejection by PI3K p110δ inhibition correlated with abrogation of antigen-specific T cell access to the transplanted heart. In contrast, loss of PI3K p110δ activity did not affect T cell priming in our system, despite evidence suggesting that this mediator is essential and non-redundant for TCR-induced activation of both naïve and memory T cells [27]. While genetic abrogation of PI3K p110δ activity might have been compensated for by alternative signaling pathways leading to T cell activation and differentiation, pharmacologic inhibition of PI3K p110δ post-priming also appears to selectively affect T cell trafficking to the heart without affecting T cell responsiveness. In the p110δ^D910A^ mouse, naive CD4^+^ T cell proliferation and cytokine production is particularly impaired under suboptimal stimulation conditions (e.g., in the absence of costimulation)[26]. It is possible that PI3K p110δ signals contributing to T cell activation might be dispensable when antigen is not limiting, such as in transplantation settings.

The role of macrophages in the development of allograft chronic rejection is still controversial [37]–[39]. In our study, macrophage infiltration was increased (while not always significantly) in both female (non-antigenic) and male heart transplants compared to native hearts even in the absence of PI3K p110δ signaling, suggesting that PI3K p110δ activity is not required for monocyte recruitment. However, macrophage infiltration did not affect the clinical outcome, suggesting that either macrophages do not contribute to tissue damage in chronic heart allograft rejection or that a cross talk with infiltrating T cells is necessary for macrophage-mediated pathologic effects.

PI3K p110δ inactivation did not affect HY-mismatched skin rejection, despite inhibiting adoptively transferred HY-specific effector T cell access to the skin graft. The immune responses against mHC antigens of skin and heart grafts have been shown to rely upon different immune mechanisms. First, HY-mismatched heart grafts develop chronic T cell infiltrates and vasculopathy over time but the organ remains viable and the heartbeat is maintained. In contrast, HY-mismatched skin grafts fail on average within 3 weeks of transplantation [28], suggesting that the graft microenvironment (size and antigen presenting cells richness) differently impacts on the strength of the alloresponse. Second, anatomical vascular connection of heart allograft to the host circulation is immediate, while connection of skin graft to the host vascular system occurs within 2–3 weeks post grafting [33], [34], [40], therefore T cell access to skin grafts is not regulated by endothelial barriers at least in early rejection. Additionally, heart graft endothelium remains of donor origin post grafting [41], [42], while skin graft re-vascularization partially relies upon cells of host origin[33], [34]. Most importantly, T cell-dependent skin graft rejection can occur acutely in an antigen-independent manner, as H-2b HY-specific, TCR transgenic Mata Hari CD8^+^ T cells can efficiently reject H-2k skin but not heart allografts [28]. The immune pathway underlying TCR-independent skin rejection has been shown to depend on IFN-γ[28], but its cellular and molecular components have not yet been identified.

Hence, while still inhibiting specific T cell trafficking into the skin, the failure of PI3K p110δ inactivation to improve skin graft survival might be due to inflammation-induced mechanisms leading to by-standing damage, related to the temporary lack of regulating endothelial barrier and possibly triggered by overwhelming host cross-reactive T cell responses against skin-harbored microbial antigens. While the heart is contained within a sterile environment inside the body, the skin is continuously exposed to environmental microorganisms.

In summary, the observations described in this study strongly support the concept that pharmacological inactivation of PI3K p110δ activity is a viable strategy to control heart allograft chronic rejection. Additional advantages of this approach include the possibility of inhibiting T-cell mediated inflammation in the context of an established immune response (i.e. after transplantation, as we have shown in this study), and the maintenance of immune reactivity, which causes severe side-effect associated with conventional immunosuppressive therapies. In this context, a PI3K p110γ?δ dual selective inhibitor has been shown to significantly reduce inflammatory injuries *in vivo* in heart ischemia-reperfusion injury models in rat and pig, while at the same time spare tissue repair processes such as EC mitogenesis [43]. Clearly, the therapeutic application of PI3K p110δ inhibition will require careful planning dictated by the organ-specific immunobiology of graft rejection. We propose that this strategy would be very effective in the context of slow-developing T cell-induced inflammation relying upon antigen-dependent trafficking including chronic rejection of vascularized tissue grafts, such as heart transplants, as well as other chronic, T-cell mediated autoimmune diseases such as type I diabetes and multiple sclerosis.

## Materials and Methods

### Ethics statement

This study was carried out in strict accordance with the Home Office recommendations and under its authority following approval by the Imperial College London/Central Biomedical Services Ethics Committee (REF. PPL 70/5872 and PPL 80/1842). All surgery was performed under anesthesia and all efforts were made to minimize suffering.

### Animals

C57BL/6 mice were purchased from Harlan Olac (Bicester, UK) and used at 7–11 weeks. p110δ^D910A^ mice were generated as previously described [26]. Experimental groups included 3–6 animals per group.

### Cell culture

Memory CD4^+^ T cells specific for the Y-chromosome encoded HY peptide epitope NAGFNSNRANSSRSS and restricted by H2-Ab [44] were obtained from WT and p110δ^D910A^ mice by two fortnightly i.p. immunizations of female mice with male C57BL/6 splenocytes, as previously described [45]. The two T cell populations displayed similar specificity, as assessed by [^3^H]thymidine incorporation, and phenotype, as established by flow cytometry (Figure S5).

### Reagents

The HY peptides encoding the *Dby* and *Uty* epitopes were kindly provided by Dr. Jian Guo Chai (Imperial College, London, UK). The PI3K p110δ inhibitor IC87114 was synthesized as described (D030 from patent WO 01/81346) [27]. IC87114 inhibits p110δ kinase activity in cells with an IC_50_ between 0.1μM and 0.5μM, and only shows cross-reactivity with other PI3K isoforms at concentrations more than 5μM [27], [46]. In vivo, IC87114 was administered i.p. at a dose of 60 mg/kg. This dose was chosen based on previous reports of its efficacy in vivo [47]. In our hands, a 30 mg/kg by gavage achieves ∼2 µM 90 min post-administration and the drug is cleared from the blood 4–7 hours post admin. IC87114 is selective for p110δ at plasma concentrations of 5 µM [47].

The cell linkers PKH26 and CFSE were purchased from Sigma-Aldrich.

### Flow cytometry

For surface staining, cells were labelled with the appropriate concentration of fluorescence-conjugated antibodies or isotype control according to the manufacturer’s instructions, and analyzed by a two-laser BD fluorescence activated cell sorter (FACS) Calibur (BD Biosciences, Oxford, UK). Acquired samples were analyzed using Flowjo 7.6 (TreeStar Inc., UK).

### T cell proliferation assays

T cells (10^4^ /well) isolated from spleen were incubated with irradiated female splenocytes (5×10^5^/well) and HY peptides *Dby*, and *Uty* (0–100nM) in 96-well flat-bottomed plates. Plate was pulsed 48 hours later with 1µCi/well [^3^H] thymidine and incubated overnight, then harvested using the Tomtec harvester 96 and filter and counted using the Wallac Microbeta counter for Windows (all from Wallac/Perkin Elmer, Buckinghamshire, UK).

### Heart transplantation

Heterotopic heart transplantation was performed in the pathogen-free facilities at Northwick Park Institute for Medical Research (NPIMR, UK) by placing the donor heart into the recipient (WT and p110δ^D910A^) sternomastoid cavity, connecting the aortal branch to the carotid artery and the pulmonary vein to the jugular vein. Before surgery, mice were given 0.25ml Saline s.c. to prevent dehydration. Anesthetic agents included Ketamine (80–100mg/kg) and Xylazine 10mg/kg. These were administered s.c. mixed in a syringe at a ratio of 2 (Ketamine):1 (Xylazine) diluted with saline 1:1. For analgesia mice were given Rimadyl (Carprofen 50mg/ml), diluted with saline 1:10 s.c.. at a dose of 5mg/kg s.c.. Analgesics were administered prior to surgery and on day one.

To assess the effect of pharmacological inhibition of PI3K p110δ activity on graft survival, WT recipients received the selective inhibitor IC87114 at 60mg/kg/day or vehicle control i.p. daily starting 7 days after transplantation and for 15 days.

At the indicated time points, all grafts and native hearts were evaluated by histopathologic criteria in a single-blinded manner (G. Stamp, Histopathology, Imperial College London) and scored to grade the degree of inflammation from 0 to 4 [48] (0, no inflammation; 1, light focal lymphohistocytic infiltrate; 2, moderate focal lymphohistocytic infiltrate with myocardial involvement; 3, moderate to severe inflammation with focal vasculopathy and myocyte degeneration; 4, severe inflammation, vasculopathy and myocardial fiber loss).

### Histochemistry

Five-micrometer-thick, paraffin-embedded sections were deparaffinized, rehydrated in graded ethanol. For elastin staining, sections were stained with Miller's elastin followed by immunoperoxidase staining for smooth muscle cells (SMCs) using rabbit monoclonal antibody to mouse SMC alpha actin (clone E184, from Epitomics, California), then counterstained with hematoxylin. For the purpose of comparison, tissue sections were taken in corresponding regions of the heart (proximal ventricular areas). Luminal occlusion was evaluated by tracing the cross-section of each vessel's internal elastic lamina and lumen using Lucia NIS elements software (Nikon UK Ltd., United Kingdom) in three transverse sections per graft. All vessels in each section, which demonstrated clear staining of elastin laminar and presence of SMC alpha-actin, were measured in three sections of each heart [48]. For immunohistochemistry, tissue sections were incubated for 1 h at room temperature with either FITC labelled anti-CD3 antibody or PE labelled anti-MAC2 antibody. Nucleus was counterstained with Vectashield mounting medium for fluorescence with DAPI (Vector Laboratories). Cell infiltration was evaluated by wide field microscopy and automated cell counting.

### Skin grafting

Skin grafting was conducted by the method of Billingham and Medawar [16] using tail skin from WT donors grafted onto the lateral thorax of either WT or p110δ^D910A^ female mice. Skin graft rejection was assessed as previously described [49]. In the experiments assessing the effect of pharmacological inhibition of p110δ activity on graft survival, WT recipients received the selective inhibitor IC87114 at 60mg/kg/day or vehicle control i.p. daily starting 7 days after transplantation and for 15 days. Prior to surgery, mice received medetomidine (1mg/kg), ketamine (75mg/kg) and atipamezole (2.5mg/kg) s.c..

### Recruitment of circulating T cells into tissues

In adoptive transfer experiments HY-specific memory T cells were incubated at 37°C for 10 minutes either with PKH26 (5 μM, red) or CFSE (1 μM, green), washed 3 times with PBS and then co-injected i.v. (10^7^/mouse). After 24 hours, mice were sacrificed and tissues were sampled and embedded in optimal cutting temperature compound (CellPath Ltd, Newtown Powys). Tissue infiltration by T cells was assessed by wide-field fluorescence microscopy 24 hours after injection. The following combinations were used: WT (red) and P110δ***^D910A^*** (green) T cells, WT T cells pre-treated with vehicle (1%DMSO, red) and with PI3K p110δ inhibitor IC87114 (5µM for 1 hour at 37°C, green).

### Wide-field fluorescence microscopy and automatic cell counting

Snap-frozen tissue sections were laid onto Polysine Microscope slides (VWR International), and then mounted in Vectashield mounting medium for fluorescence with DAPI (Vector Laboratories), to stain the nuclei. Slides were visualized with a Coolview 12-cooled CCD camera (Photonic Science) mounted over a Zeiss Axiovert S100 microscope equipped with Metamorph software (Zeiss). Tissue infiltration was quantified by randomly selecting ten ×10-magnified fields from tissue samples from at least 3 animals and assessing the number of fluorescent cells in each field. Quantification of T cell infiltrates observed by wide-field fluorescence microscopy was performed using a specifically designed software to run in the LabView (version 7.1; National Instruments) environment. This automatic cell counting algorithm is based on a combination of background subtraction, multiple thresholding, and morphological processing approaches [50], which allow identification of single fluorescent cells within the tissue. The number of infiltrating labelled cells were then averaged and assessed statistically. Infiltration is expressed as the mean of fluorescent cells per ×10 field in a given experimental condition ± SD.

### Statistics

Results are given as the mean per group ± SD. The data were analyzed using a two-tailed unpaired Student’s t test and Mann-Whitney test. A P value of less than 0.05 was considered significant.

## Supporting Information

Figure S1 **Histology of transplanted and native hearts.** Recipient female WT and p110δ^D910A^ mutant mice received either male or female WT hearts. 23 days after transplant, both transplanted and native hearts were harvested and stained with hematoxilin/eosin. Each panel shows a representative tissue image. Magnification: 20x.
(DOC)

Figure S2 **Immunohistochemistry of transplanted and native hearts**. Recipient female WT mice received either syngeneic male or female heart grafts. After 7 days, the selective PI3K p110δ inhibitor IC87114 (60mg/kg/day) or vehicle control were injected i.p. daily for 15 days. Mice were sacrificed 24 hours after the last treatment (day 23). (A) Both transplanted and native hearts were harvested and stained with hematoxilin/eosin. Each panel shows a representative tissue image. Magnification: 20x. (B) Both transplanted and native hearts were harvested and tissue sections were stained with either FITC-labelled anti-CD3 antibody or PE-labelled anti-MAC2 antibody. Each panel shows a representative tissue image. Magnification: 20x.
(DOC)

Figure S3 **Pharmacologic inactivation of PI3K p110**δ **does not induce T cell tolerance.** Recipient female WT mice received either syngeneic male or female heart grafts. After 7 days, the selective PI3K p110δ inhibitor IC87114 (60mg/kg/day) or vehicle control were injected i.p. daily for 15 days. Mice were sacrificed 24 hours after the last treatment (day 23). Splenocytes obtained from WT female recipients treated with or without IC87114 were incubated with different concentrations of *Dby* and *Uty* HY peptide epitopes for 48 hours, followed by pulsing with [^3^H] thymidine to assess T cell proliferation.
(DOC)

Figure S4 **Genetic or pharmacologic inactivation of PI3K p110δ do not induce T cell tolerance in recipients of skin allografts.** (A) Recipient female WT and p110δ^D910A^ mutant mice received male skin grafts. After skin grafts were rejected, splenocytes from recipient mice were harvested and incubated with different concentrations of *Dby* and *Uty* HY epitopes for 48 hours, followed by pulsing with [^3^H] thymidine to assess T cell proliferation. (B) Recipient female WT mice received male skin grafts. 7 days after transplant, the PI3K p110δ inhibitor IC87114 at 60mg/kg/day or vehicle control were injected i.p. daily until the grafts were rejected. Splenocytes from recipient mice were harvested and incubated with different concentrations of *Dby* and *Uty* HY epitopes for 48 hours, followed by pulsing with [^3^H] thymidine to assess T cell proliferation. Filled symbols: *Dby*; Empty symbols: *Uty*.
(DOC)

Figure S5 **Characterization of HY-specific WT and p110δ^D910A^ T cells**. (A) HY-specific CD4^+^ WT and p110δ^D910A^ T cells were harvested between days seven and ten post-stimulation with irradiated male splenocytes. T cells were stained with monoclonal antibodies recognizing CD4, CD8, CD62L and CCR7 and appropriate isotype control antibodies and analysed by flow cytometry. Expression of CD4, CD8, CD62L and CCR7 is shown in bold while the dotted line represents the isotype control. (B) WT or p110δ^D910A^ T cells were incubated with 6 x10^6^ female irradiated splenocytes and different concentrations of *Dby* (filled symbols) and *Uty* (empty symbols) HY epitopes for 48 hours, followed by pulsing with [^3^H] thymidine to assess proliferation.
(DOC)

## References

[1] Tanaka M, Fedoseyeva EV, Robbins RC (2005). Graft coronary artery disease in murine cardiac allografts: proposal to meet the need for standardized assessment.. JHeart Lung Transplant.

[2] Taylor DO, Edwards LB, Mohacsi PJ, Boucek MM, Trulock EP (2003). The registry of the International Society for Heart and Lung Transplantation: twentieth official adult heart transplant report--2003.. JHeart Lung Transplant.

[3] Taylor DO, Stehlik J, Edwards LB, Aurora P, Christie JD (2009). Registry of the International Society for Heart and Lung Transplantation: Twenty-sixth Official Adult Heart Transplant Report-2009.. J Heart Lung Transplant.

[4] van Loosdregt J, van Oosterhout MF, Bruggink AH, van Wichen DF, van Kuik J (2006). The chemokine and chemokine receptor profile of infiltrating cells in the wall of arteries with cardiac allograft vasculopathy is indicative of a memory T-helper 1 response.. Circulation.

[5] Lee RS, Yamada K, Houser SL, Womer KL, Maloney ME (2001). Indirect recognition of allopeptides promotes the development of cardiac allograft vasculopathy.. ProcNatlAcadSciUSA.

[6] Libby P, Pober JS (2001). Chronic rejection.. Immunity.

[7] Liu Z, Colovai AI, Tugulea S, Reed EF, Fisher PE (1996). Indirect recognition of donor HLA-DR peptides in organ allograft rejection.. JClinInvest.

[8] Waaga AM, Gasser M, Laskowski I, Tilney NL (2000). Mechanisms of chronic rejection.. CurrOpinImmunol.

[9] Chen Y, Demir Y, Valujskikh A, Heeger PS (2003). The male minor transplantation antigen preferentially activates recipient CD4+ T cells through the indirect presentation pathway in vivo.. JImmunol.

[10] He C, Schenk S, Zhang Q, Valujskikh A, Bayer J (2004). Effects of T cell frequency and graft size on transplant outcome in mice.. JImmunol.

[11] Huddleston SJ, Hays WS, Filatenkov A, Ingulli E, Jenkins MK (2006). CD154+ graft antigen-specific CD4+ T cells are sufficient for chronic rejection of minor antigen incompatible heart grafts.. AmJTransplant.

[12] Schnickel GT, Whiting D, Hsieh GR, Yun JJ, Fischbein MP (2004). CD8 lymphocytes are sufficient for the development of chronic rejection.. Transplantation.

[13] Sun H, Woodward JE, Subbotin VM, Kuddus R, Logar AJ (2002). Use of recombinase activation gene-2 deficient mice to ascertain the role of cellular and humoral immune responses in the development of chronic rejection.. JHeart Lung Transplant.

[14] Szeto WY, Krasinskas AM, Kreisel D, Krupnick AS, Popma SH (2002). Depletion of recipient CD4+ but not CD8+ T lymphocytes prevents the development of cardiac allograft vasculopathy.. Transplantation.

[15] Valujskikh A, Zhang Q, Heeger PS (2006). CD8 T cells specific for a donor-derived, self-restricted transplant antigen are nonpathogenic bystanders after vascularized heart transplantation in mice.. JImmunol.

[16] Billingham RE, Brent L, Medawar PB (1953). Actively acquired tolerance of foreign cells.. Nature.

[17] Kawai T, Shimauchi H, Eastcott JW, Smith DJ, Taubman MA (1998). Antigen direction of specific T-cell clones into gingival tissues.. Immunology.

[18] Marelli-Berg FM, Frasca L, Weng L, Lombardi G, Lechler RI (1999). Antigen recognition influences transendothelial migration of CD4+ T cells.. JImmunol.

[19] Marelli-Berg FM, James MJ, Dangerfield J, Dyson J, Millrain M (2004). Cognate recognition of the endothelium induces HY-specific CD8+ T-lymphocyte transendothelial migration (diapedesis) in vivo.. Blood.

[20] Savinov AY, Wong FS, Stonebraker AC, Chervonsky AV (2003). Presentation of antigen by endothelial cells and chemoattraction are required for homing of insulin-specific CD8+ T cells.. JExpMed.

[21] Jarmin SJ, David R, Ma L, Chai JG, Dewchand H (2008). T cell receptor-induced phosphoinositide-3-kinase p110delta activity is required for T cell localization to antigenic tissue in mice.. JClinInvest.

[22] Sobel RA, Blanchette BW, Bhan AK, Colvin RB (1984). The immunopathology of experimental allergic encephalomyelitis. II. Endothelial cell Ia increases prior to inflammatory cell infiltration.. J Immunol.

[23] Okkenhaug K, Vanhaesebroeck B (2003). PI3K in lymphocyte development, differentiation and activation.. NatRevImmunol.

[24] Wang J, Auger KR, Jarvis L, Shi Y, Roberts TM (1995). Direct association of Grb2 with the p85 subunit of phosphatidylinositol 3-kinase.. JBiolChem.

[25] Zhang W, Sloan-Lancaster J, Kitchen J, Trible RP, Samelson LE (1998). LAT: the ZAP-70 tyrosine kinase substrate that links T cell receptor to cellular activation.. Cell.

[26] Okkenhaug K, Bilancio A, Farjot G, Priddle H, Sancho S (2002). Impaired B and T cell antigen receptor signaling in p110delta PI 3-kinase mutant mice.. Science.

[27] Soond DR, Bjorgo E, Moltu K, Dale VQ, Patton DT (2010). PI3K p110delta regulates T-cell cytokine production during primary and secondary immune responses in mice and humans.. Blood.

[28] Valujskikh A, Lantz O, Celli S, Matzinger P, Heeger PS (2002). Cross-primed CD8(+) T cells mediate graft rejection via a distinct effector pathway.. NatImmunol.

[29] Simpson E, Scott D, Chandler P (1997). The male-specific histocompatibility antigen, H-Y: a history of transplantation, immune response genes, sex determination and expression cloning.. Annu Rev Immunol.

[30] Amano J, Ishiyama S, Nishikawa T, Tanaka H, Nagai R (1997). Proliferation of smooth muscle cells in acute allograft vascular rejection.. J Thorac Cardiovasc Surg.

[31] Reif K, Okkenhaug K, Sasaki T, Penninger JM, Vanhaesebroeck B (2004). Cutting edge: differential roles for phosphoinositide 3-kinases, p110gamma and p110delta, in lymphocyte chemotaxis and homing.. J Immunol.

[32] Kearney ER, Pape KA, Loh DY, Jenkins MK (1994). Visualization of peptide-specific T cell immunity and peripheral tolerance induction in vivo.. Immunity.

[33] de Waal RM, Bogman MJ, Cornelissen IM, Vermeulen AN, Koene RA (1986). Expression of donor class I major histocompatibility antigens on the vascular endothelium of mouse skin allografts.. Transplantation.

[34] de Waal RM, Bogman MJ, Maass CN, Cornelissen LM, Tax WJ (1983). Variable expression of Ia antigens on the vascular endothelium of mouse skin allografts.. Nature.

[35] Marelli-Berg FM, Cannella L, Dazzi F, Mirenda V (2008). The highway code of T cell trafficking.. JPathol.

[36] Manes TD, Pober JS (2008). Antigen presentation by human microvascular endothelial cells triggers ICAM-1-dependent transendothelial protrusion by, and fractalkine-dependent transendothelial migration of, effector memory CD4+ T cells.. JImmunol.

[37] Christen T, Nahrendorf M, Wildgruber M, Swirski FK, Aikawa E (2009). Molecular imaging of innate immune cell function in transplant rejection.. Circulation.

[38] Murase N, Ichikawa N, Ye Q, Chun HJ, Okuda T (1999). Dendritic cells/chimerism/alleviation of chronic allograft rejection.. JLeukocBiol.

[39] Ozdemir BH, Sezgin A, Haberal M (2009). Apoptosis and proliferation of cardiomyocytes and interstitial mononuclear cells: association with rejection and macrophage infiltration.. TransplantProc.

[40] Capla JM, Ceradini DJ, Tepper OM, Callaghan MJ, Bhatt KA (2006). Skin graft vascularization involves precisely regulated regression and replacement of endothelial cells through both angiogenesis and vasculogenesis.. PlastReconstrSurg.

[41] Hasegawa S, Becker G, Nagano H, Libby P, Mitchell RN (1998). Pattern of graft- and host-specific MHC class II expression in long-term murine cardiac allografts: origin of inflammatory and vascular wall cells.. AmJPathol.

[42] Quaini F, Urbanek K, Beltrami AP, Finato N, Beltrami CA (2002). Chimerism of the transplanted heart.. NEnglJMed.

[43] Doukas J, Wrasidlo W, Noronha G, Dneprovskaia E, Fine R (2006). Phosphoinositide 3-kinase gamma/delta inhibition limits infarct size after myocardial ischemia/reperfusion injury.. ProcNatlAcadSciUSA.

[44] Scott D, Addey C, Ellis P, James E, Mitchell MJ (2000). Dendritic cells permit identification of genes encoding MHC class II-restricted epitopes of transplantation antigens.. Immunity.

[45] Millrain M, Chandler P, Dazzi F, Scott D, Simpson E (2001). Examination of HY response: T cell expansion, immunodominance, and cross-priming revealed by HY tetramer analysis.. JImmunol.

[46] Knight ZA, Gonzalez B, Feldman ME, Zunder ER, Goldenberg DD (2006). A pharmacological map of the PI3-K family defines a role for p110alpha in insulin signaling.. Cell.

[47] Ali K, Bilancio A, Thomas M, Pearce W, Gilfillan AM (2004). Essential role for the p110delta phosphoinositide 3-kinase in the allergic response.. Nature.

[48] Xu Y, Chester AH, Hariri B, McCormack A, Sarathchandra P (2010). The indirect alloimmune response causes microvascular endothelial dysfunction-a possible role for alloantibody.. Transplantation.

[49] Schwoebel F, Barsig J, Wendel A, Hamacher J (2005). Quantitative assessment of mouse skin transplant rejection using digital photography.. Lab Anim.

[50] Mirenda V, Jarmin SJ, David R, Dyson J, Scott D (2007). Physiologic and aberrant regulation of memory T-cell trafficking by the costimulatory molecule CD28.. Blood.

