# Supplementary material for: Genetic or Pharmaceutical Blockade of Phosphoinositide 3-Kinase P110δ Prevents Chronic Rejection of Heart Allografts
Source: PLoS One. 2012 Mar 30;7(3):e32892. doi: 10.1371/journal.pone.0032892 (PMC3316549; doi:10.1371/journal.pone.0032892)
Supplement: Figure S1 — Histology of transplanted and native hearts. Recipient female WT and p110δD910A mutant mice received either male or female WT hearts. 23 days after transplant, both transplanted and native hearts were harvested and stained with hematoxilin/eosin. Each panel shows a representative tissue image. Magnification: 20x. (DOC) [file pone.0032892.s001.doc]

***Figure S1***

***Histology of transplanted and native hearts.***

Recipient female WT and p110δ^D910A^ mutant mice received either male or female WT hearts. 23 days after transplant, both transplanted and native hearts were harvested and stained with hematoxilin/eosin. Each panel shows a representative tissue image. Magnification: 20x.
