# Supplementary material for: Genetic or Pharmaceutical Blockade of Phosphoinositide 3-Kinase P110δ Prevents Chronic Rejection of Heart Allografts
Source: PLoS One. 2012 Mar 30;7(3):e32892. doi: 10.1371/journal.pone.0032892 (PMC3316549; doi:10.1371/journal.pone.0032892)
Supplement: Figure S2 — Immunohistochemistry of transplanted and native hearts. Recipient female WT mice received either syngeneic male or female heart grafts. After 7 days, the selective PI3K p110δ inhibitor IC87114 (60mg/kg/day) or vehicle control were injected i.p. daily for 15 days. Mice were sacrificed 24 hours after the last treatment (day 23). (A) Both transplanted and native hearts were harvested and stained with hematoxilin/eosin. Each panel shows a representative tissue image. Magnification: 20x. (B) Both transplanted and native hearts were harvested and tissue sections were stained with either FITC-labelled anti-CD3 antibody or PE-labelled anti-MAC2 antibody. Each panel shows a representative tissue image. Magnification: 20x. (DOC) [file pone.0032892.s002.doc]

***Figure S2***

**ABnd native heart 1B and Figure 1C in this paragraph000000000000000000000000000000000000000000000000000000000000000000000000000**

**B**

***Immunohistochemistry of transplanted and native hearts***.

Recipient female WT mice received either syngeneic male or female heart grafts. After 7 days, the selective PI3K p110δ inhibitor IC87114 (60mg/kg/day) or vehicle control were injected i.p. daily for 15 days. Mice were sacrificed 24 hours after the last treatment (day 23). **(A)** Both transplanted and native hearts were harvested and stained with hematoxilin/eosin. Each panel shows a representative tissue image. Magnification: 20x. **(B)** Both transplanted and native hearts were harvested and tissue sections were stained with either FITC-labelled anti-CD3 antibody or PE-labelled anti-MAC2 antibody. Each panel shows a representative tissue image. Magnification: 20x.
