# Supplementary material for: Genetic or Pharmaceutical Blockade of Phosphoinositide 3-Kinase P110δ Prevents Chronic Rejection of Heart Allografts
Source: PLoS One. 2012 Mar 30;7(3):e32892. doi: 10.1371/journal.pone.0032892 (PMC3316549; doi:10.1371/journal.pone.0032892)
Supplement: Figure S3 — Pharmacologic inactivation of PI3K p110δ does not induce T cell tolerance. Recipient female WT mice received either syngeneic male or female heart grafts. After 7 days, the selective PI3K p110δ inhibitor IC87114 (60mg/kg/day) or vehicle control were injected i.p. daily for 15 days. Mice were sacrificed 24 hours after the last treatment (day 23). Splenocytes obtained from WT female recipients treated with or without IC87114 were incubated with different concentrations of Dby and Uty HY peptide epitopes for 48 hours, followed by pulsing with [3H] thymidine to assess T cell proliferation. (DOC) [file pone.0032892.s003.doc]

***Figure S3***

***Pharmacologic inactivation of PI3K p110***δ ***does not induce T cell tolerance.***

Recipient female WT mice received either syngeneic male or female heart grafts. After 7 days, the selective PI3K p110δ inhibitor IC87114 (60mg/kg/day) or vehicle control were injected i.p. daily for 15 days. Mice were sacrificed 24 hours after the last treatment (day 23). Splenocytes obtained from WT female recipients treated with or without IC87114 were incubated with different concentrations of *Dby* and *Uty* HY peptide epitopes for 48 hours, followed by pulsing with [^3^H] thymidine to assess T cell proliferation.
