# Supplementary material for: Genetic or Pharmaceutical Blockade of Phosphoinositide 3-Kinase P110δ Prevents Chronic Rejection of Heart Allografts
Source: PLoS One. 2012 Mar 30;7(3):e32892. doi: 10.1371/journal.pone.0032892 (PMC3316549; doi:10.1371/journal.pone.0032892)
Supplement: Figure S4 — Genetic or pharmacologic inactivation of PI3K p110δ do not induce T cell tolerance in recipients of skin allografts. (A) Recipient female WT and p110δD910A mutant mice received male skin grafts. After skin grafts were rejected, splenocytes from recipient mice were harvested and incubated with different concentrations of Dby and Uty HY epitopes for 48 hours, followed by pulsing with [3H] thymidine to assess T cell proliferation. (B) Recipient female WT mice received male skin grafts. 7 days after transplant, the PI3K p110δ inhibitor IC87114 at 60mg/kg/day or vehicle control were injected i.p. daily until the grafts were rejected. Splenocytes from recipient mice were harvested and incubated with different concentrations of Dby and Uty HY epitopes for 48 hours, followed by pulsing with [3H] thymidine to assess T cell proliferation. Filled symbols: Dby; Empty symbols: Uty. (DOC) [file pone.0032892.s004.doc]

***Figure S4***

***Genetic or pharmacologic inactivation of PI3K p110δ do not induce T cell tolerance in recipients of skin allografts.***

**(A)** Recipient female WT and p110δ^D910A^ mutant mice received male skin grafts. After skin grafts were rejected, splenocytes from recipient mice were harvested and incubated with different concentrations of *Dby* and *Uty* HY epitopes for 48 hours, followed by pulsing with [^3^H] thymidine to assess T cell proliferation. **(B)** Recipient female WT mice received male skin grafts. 7 days after transplant, the PI3K p110δ inhibitor IC87114 at 60mg/kg/day or vehicle control were injected i.p. daily until the grafts were rejected. Splenocytes from recipient mice were harvested and incubated with different concentrations of *Dby* and *Uty* HY epitopes for 48 hours, followed by pulsing with [^3^H] thymidine to assess T cell proliferation. Filled symbols: *Dby*; Empty symbols: *Uty*.
