# Supplementary material for: Genetic or Pharmaceutical Blockade of Phosphoinositide 3-Kinase P110δ Prevents Chronic Rejection of Heart Allografts
Source: PLoS One. 2012 Mar 30;7(3):e32892. doi: 10.1371/journal.pone.0032892 (PMC3316549; doi:10.1371/journal.pone.0032892)
Supplement: Figure S5 — Characterization of HY-specific WT and p110δD910A T cells. (A) HY-specific CD4+ WT and p110δD910A T cells were harvested between days seven and ten post-stimulation with irradiated male splenocytes. T cells were stained with monoclonal antibodies recognizing CD4, CD8, CD62L and CCR7 and appropriate isotype control antibodies and analysed by flow cytometry. Expression of CD4, CD8, CD62L and CCR7 is shown in bold while the dotted line represents the isotype control. (B) WT or p110δD910A T cells were incubated with 6 x10^6^ female irradiated splenocytes and different concentrations of Dby (filled symbols) and Uty (empty symbols) HY epitopes for 48 hours, followed by pulsing with [3H] thymidine to assess proliferation. (DOC) [file pone.0032892.s005.doc]

***Figure S5***

***Characterization of WT and p110δ^D910A^ T cells.***

**(A)** HY-specific CD4^+^ WT and p110δ^D910A^ T cells were harvested between days seven and ten post-stimulation with irradiated male splenocytes. T cells were stained with monoclonal antibodies recognizing CD4, CD8, CD62L and CCR7 and appropriate isotype control antibodies and analysed by flow cytometry. Expression of CD4, CD8, CD62L and CCR7 is shown in bold while the dotted line represents the isotype control. **(B)** WT or p110δ^D910A^ T cells were incubated with 6 x10^6^ female irradiated splenocytes and different concentrations of *Dby* (filled symbols) and *Uty* (empty symbols) HY epitopes for 48 hours, followed by pulsing with [^3^H] thymidine to assess proliferation.
